# History of the Strange Sounds or Rappings, Heard in Rochester and Western New York, and Usually Called the Mysterious Noises, &c.

**Published:** 1851-03

**Authors:** 


### REVIEWS.

---

ART. I.—*History of the Strange Sounds or Rappings, heard in Rochester and Western New York, and usually called the Mysterious Noises, &c.* By D. M. DEWEY, Rochester, 1850.

*Exposition of the Rochester Knockings.* By AUSTIN FLINT, M. D., CHARLES A. LEE, M. D., and C. B. COVENTRY, M. D. Buffalo Commercial Advertiser, Feb. 17th, 1851.

The newspaper press has teemed for many months past with marvelous accounts of strange noises, heard in various towns of western New York and held by the superstitious to be connected with the spiritual world. These narrations have excited the most intense interest in the popular mind. Persons from all parts of the country have visited Rochester to listen to the mysterious rappings, and have given to the public the most wonderful descriptions of what they saw and heard. Mesmerism has been thrown into the shade; the feats of the "Phreno-Magnetisers" have been beggared by the exploits of the Rochester "witches." Hitherto we have refrained from all notice of this affair, because we felt satisfied that it was a delusion, and that in time it would meet with a just exposure. An exposition of the mystery has been made, and we purpose now, with the explanation, to give a brief history of the imposture. In doing so, let us not be accused of traveling beyond the bounds of our profession—our readers will see, before we are done, that the subject is one which comes legitimately within the province of physiology.

The females who have attracted so much attention are Mrs. Fish, a widow, and Miss Margaretta Fox, and Miss Catherine Fox, her sisters, young girls, aged, one fourteen and the other sixteen years. They have been considered respectable people. At first, their exhibitions were free to all the curious, but for some time past they have been perambulating the country fleecing the gullible of their dollars. In the house of their father, Mr. John D. Fox, the noises were first heard about the latter end of March, 1848, one evening after the family had retired for the night. They seemed to be in one of the bedrooms, and sounded as though some one was knocking on the floors, moving chairs, etc. A perceptible jar was felt by the persons present when their hands were placed on the furniture in the room, and was also experienced by them as they stood on the floor. This has been constantly observed ever since in all the exhibitions of these wonder-mongers. The Rev. C. Hammond, of Rochester, undertook to investigate the strange phenomena, and the following is an extract from his letter describing the manifestations of one evening:—

"On taking our positions the sounds were heard, and continued to multiply and become more violent, until every part of the room trembled with their demonstrations. They were unlike any I had heard before. Suddenly, as we were all resting on the table, I felt the side next to me move upward—I pressed upon it heavily, but soon it passed out of the reach of us all—full six feet from me, and at least four from the nearest person to it. I saw distinctly its position—not a thread could have connected it with any of the company without my notice, for I had come to detect imposition, if it could be found. In this position we were situated, when the question was asked, "Will the spirit move the table back where it stood before?"—And back it came, as though it were carried on the head of some one, who had not suited his position to a perfect equipoise, the balance being sometimes in favor of one side, and then the other. But it regained its first position. In the mean time the "demonstrations" grew louder and louder. The family commenced and sung the "spirit's song," and several other pieces of sacred music, during which accurate time was marked on the table, causing it to vibrate—a transparent hand, resembling a shadow, presented itself before my face—I felt fingers taking hold of a lock of hair on the left side of my head, causing an inclination of several inches—then a cold, death-like hand was drawn designedly over my face—three gentle raps on my left knee—my right limb forcibly pulled, against strong resistance, under the table—a violent shaking, as though two hands were applied to my shoulders—myself and chair uplifted and moved back a few inches, and several slaps, as with a hand, on the side of my head, which were repeated on each one of the company, more rapid than I could count. During these manifestations, a piece of pasteboard, nearly a foot square, was swung with such velocity before us as to throw a strong current of air in our faces—a paper curtain attached to one of the windows was rolled up and unrolled twice—a lounge immediately behind me was shaken violently—two small drawers in a bureau, played back and forth with inconceivable rapidity—a sound resembling a man sawing boards, and planing them, was heard under the table—a common spinning-wheel seemed to be in motion, making a very natural buzz of the spindle—a reel articulated each knot wound upon it, while the sound of a rocking cradle indicated maternal care for the infant's slumbers. These were among many other demonstrations which I witnessed that evening, amid which I felt a perfect self-possession, and in no instance the slightest embarrassment, except a momentary chill when the cold hand was applied to my face, similar to a sensation I have realized when touching a dead body. That any of the company could have performed these things, under the circumstances in which we were situated, would require a greater stretch of credulity on my part, than it would to believe that it was the work of spirits. It could not, by any possibility, have been done by them, nor even attempted, without detection. And I may add, that near the close of the demonstrations at this visit, there was a vibration of the floor, as though several tons in weight had been uplifted and suddenly fallen again upon it. This caused everything in the room to shake most violently for several minutes, when the force was withdrawn."

The reader will bear in mind that this is the grave statement of a serious man who writes like a scholar. He is accredited a man of veracity or his letter would not have been published, and it is manifest that he is fully persuaded of the reality of what he saw, as well as that all the commotion in the room was the work of "spirits."

The following colloquy is reported as having taken place between the "spirits" and a Mr. "P.," who had frequently been present at the performances of the witches:

"At one time, when some of these sounds had been unusually loud, I inquired the object of them. The answer was, "To convince you." I said, "You cannot be spirits, for according to my theory, spirits can pervade matter, and pass through it, but cannot move it, and handle it, as this table has been moved." Reply. "You are mistaken: we can and do affect matter at our will." "I have heard it said that you sometimes manifest yourselves to persons as by the touch of the hand, etc. Is it so?" "It is." "But it is said that you make such manifestations in the dark only; this leads to suspicions, with all the precaution that can be taken. Why not do this in the light?" "Because, that in such manifestations we assume a material form, and it would frighten. We do not wish to alarm, but to convince."

By the agency of these "spirits," a father and mother were permitted to converse with a departed child; a brother talked with his sister about the disease of which "her body died;" a man was told his age, his wife's age, "and that of every child in his family correct to a day, a thing he could not do himself," etc. On one occasion a gentleman fancied that he might catch Miss Fox by a trick. "So he thought of the name of a young lady who had been dead for several years. He asked if the "spirit" would rap on his touching the point of his pencil upon the name he was thinking of, provided he would write down ten names, and that among the ten." There was an affirmative reply. Ten names were written, but the name of the young lady was not among them. He went through the list, pointing at them successively; but there was no rap. The company seemed much astonished. He then added two more names, one being that of the young lady, "and as quick as he touched it, there came a rap." He then asked it (*mentally*, it is reported) "to spell out the name he thought of, and it spelled out the name of the young lady."

But enough. We must not trespass too far upon the patience and good nature of our readers, but we beg leave respectfully to submit to them, whether *clairvoyance* or *prevoyance* furnishes among their marvels anything more wonderful than this? And now let us see what becomes of it all.

The Rochester sorceresses had begun to turn their art to a good account. They had just concluded a profitable exhibition in the city of N. York, and had gone to Buffalo to turn the city upside down. Our friends, Professors Flint, Lee, and Coventry, of the University of Buffalo, were led by curiosity to visit their room, and while watching their movements discovered a clue to the mystery. They saw from the countenance of Miss Fox that the sounds were due to her agency, and that they involved an effort of the will. In fine, they became satisfied that they were produced *by a partial dislocation of the knee joint*, which the young woman was able to effect at pleasure. Resting her foot upon a solid support, she was able to displace the tibia, which was attended by a loud noise, and the return of the bone to its natural position gave rise to a second sound. It was a singular coincidence that furnished the gentlemen with the means of demonstrating the correctness of their explanation. After they had arrived at their conclusion, they met with a highly respectable lady in Buffalo, who, by this motion of the tibia upon the femur, can "develop sounds similar both in character and degree to those professedly elicited by the Rochester impostors from the spiritual world."

One would suppose that this would have put an end to the imposture, but the spirits were not to be laid so easily. Mrs. Fish and Miss Fox appeared next day in a "card," denying that they were impostors, and bantering their accusers to make trial of the truth of their theory. Accordingly an investigation was undertaken, the result of which is given in a letter from Dr. Lee to the editor of the New York *Tribune,* which was published on the 25th of February. Dr. Lee, in company with his colleagues, Dr. Flint, and Dr. Coventry, attended at the rooms of the ladies on the evening of the 18th of that month. Besides these gentlemen, some eight or ten individuals, including three ladies, friends of the "witches," were present by invitation. The preliminaries being arranged, it was asked whether the "spirits" would be present and communicate with the committee through the evening. After an interval of a minute or so, says Dr. Lee, raps were heard and continued in quick succession for some time, which Mrs. Fish declared to be an affirmative answer. This was repeated, so that there would be no mistake as to the willingness on the part of the "spirits" to accommodate the gentlemen.

This being fully understood, the ladies were requested to be seated on chairs, their limbs extended, and their heels resting on cushions, in which position, without a solid support to serve at once as a *fulcrum* and a medium for conveying the vibrations, it was evident, if the theory proposed was sound, there could be no noises produced. The witches remained fifty minutes in this position, during which time *no raps were heard,* though the "spirits" were urged to manifest themselves; but the moment the ladies placed their feet on the floor the knockings commenced and continued for several minutes.

But there was still another test to which the necromancers were doomed to be subjected, and this we give in Dr. Lee's own words:

Seating ourselves before the ladies, we grasped each of their knees firmly, so as to prevent any lateral movement of the bones; the "raps" immediately ceased, and were not heard while the knees were thus held, except near the close of the experiment, which continued once forty minutes, when two slight sounds were heard, on slightly relaxing my grasp, while at the same time I distinctly felt the heads of the bones grating on each other, and the muscles contracting, which, though a very positive kind of evidence to me, I am aware is not so satisfactory to bystanders.

Some times, in the course of the trial, the hands of the operators were released, and raps "were always heard during the interval of removal." At the close of the sittings, we are told by Dr. Lee, Miss Fox was much affected, and shed many tears, which excited much sympathy on the part of some of the gentlemen present.

What plea will the "witches" and their allies, the *clairvoyants*, set up? How will they account for their failure to evoke the "spirits" when their knees were held? Will they allege, as we have heard mesmerisers do, when the bandage was drawn a little too securely over the eyes of their subjects, "that the pressure disturbed the magnetic influence?" Or will they say, as a witty friend of ours suggests, "that the modest 'spirits' were frightened away at seeing the hands of the doctors on the ladies' knees?"

If we are not mistaken, there is a lesson taught by this imposture, which will not be without its effect. Here we have seen done by alleged "spiritual agency," all that is professed to be done by the subjects of clairvoyance, and after all it turns out that the revelations which have so astonished preachers and editors, and made multitudes stare wherever they have been reported, come from a clever girl's knee joint. If clairvoyance is able to hold up its head for a good while after such an exposure, we confess it will surprise us.

This Rochester imposture has been productive of much mischief; more than one individual has been driven by it to madness. A murder and a suicide have been traced to its baleful influence. The editor of the *New York Medical Gazette* mentions a case of insanity which had just occurred in that city, "by reason of the revelations made by mysterious raps, that the steamship Atlantic had been wrecked, with the loss of all on board." Among the passengers whose "spirits" were declared to have made the rapping, one has arrived at home "to find his wife a maniac, from a belief in these ghostly knockings." Mesmerism is not less pernicious. The editor mentions the case of another female who "has just been sent to the asylum, by reason of mesmeric operations upon her nervous system, avowedly for the purpose of rendering her clairvoyant, but with the effect of dooming her to lunacy. "And," he adds, "these recent instances are not mere isolated cases, for in several of the asylums the victims of these kindred impostures are hopelessly insane."

We have adverted to this subject in the hope of exciting the profession to a more united opposition to the host of impostures with which our land is infested. Physicians are the constituted guardians of the public health. They are expected to give direction to popular opinion on all matters relating to the human economy. Few of them, it is pleasant to know, were made dupes to the Rochester imposition, as few, compartively, have embraced the absurdities of mesmerism. While one, here and there, has been found with credulity sufficient to swallow all the monstrosities of clairvoyance, the great body of the profession has listened to its reputed miracles with becoming indifference. Medical men, accustomed as they are to the investigation of physical phenomena, ought to be the last, and as a general rule they have been the last, to fall into the wiles of impostors. The physiological peculiarity in the case of Miss Fox is interesting, and, from the fact that it has been found to exist in another lady in a neighboring city, is probably not so rare as might have been supposed. It is altogether probable that we shall now hear of many analogous instances.

